# Supplementary material for: Structure of putative tumor suppressor ALDH1L1
Source: Commun Biol. 2022 Jan 10;5:3. doi: 10.1038/s42003-021-02963-9 (PMC8748788; doi:10.1038/s42003-021-02963-9)
Supplement: Supplementary file 2 — Supplemental Information [file 42003_2021_2963_MOESM2_ESM.pdf]

Supplementary Information

Structure of putative tumor suppressor ALDH1L1

Yaroslav Tsybovsky, Valentin Sereda, Marcin Golczak, Natalia I. Krupenko, Sergey A. Krupenko

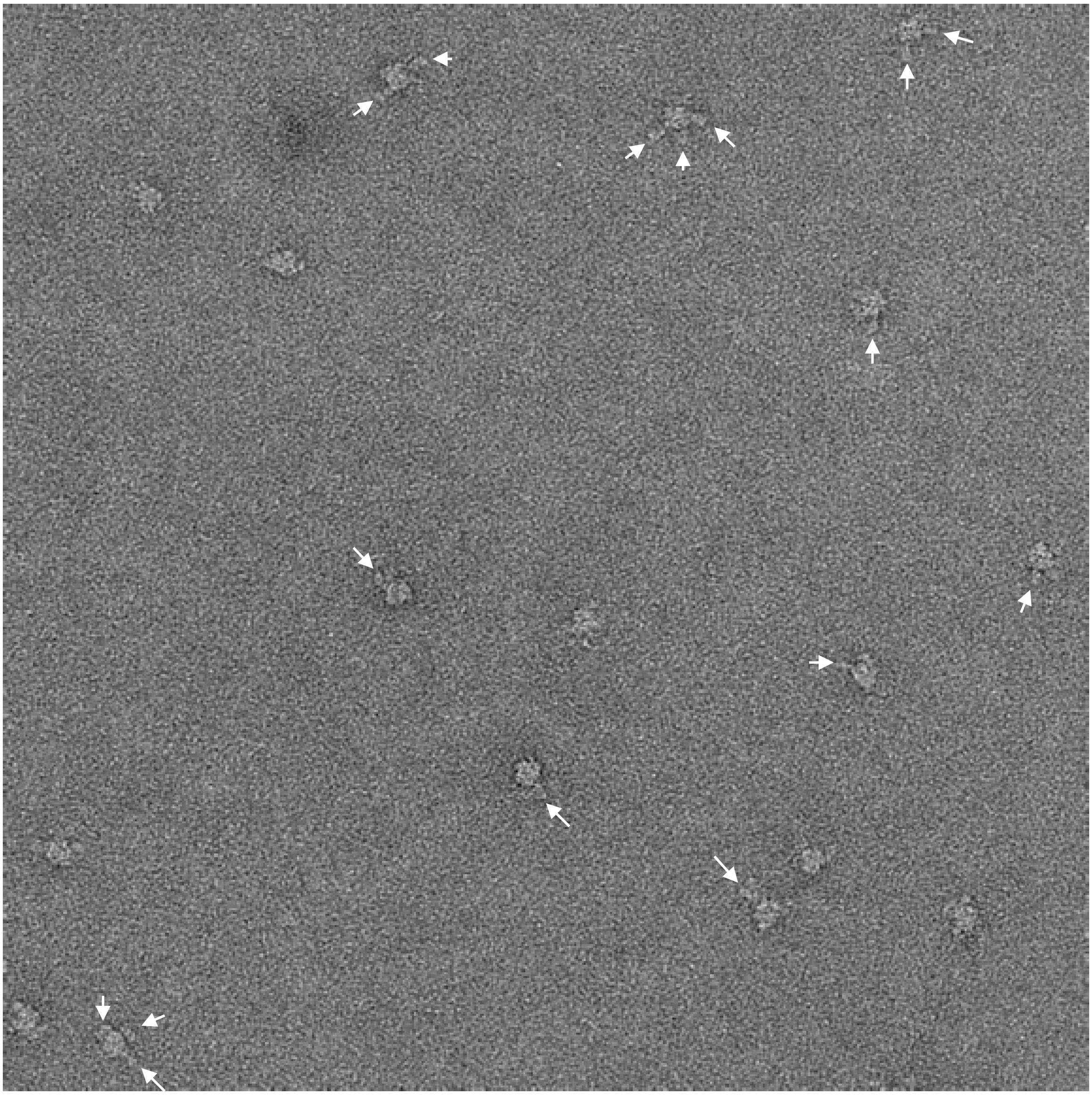

Supplementary Figure 1. A representative micrograph of ALDH1L1 negatively stained with uranyl formate. Arrows point to clearly discernible N-terminal domains.

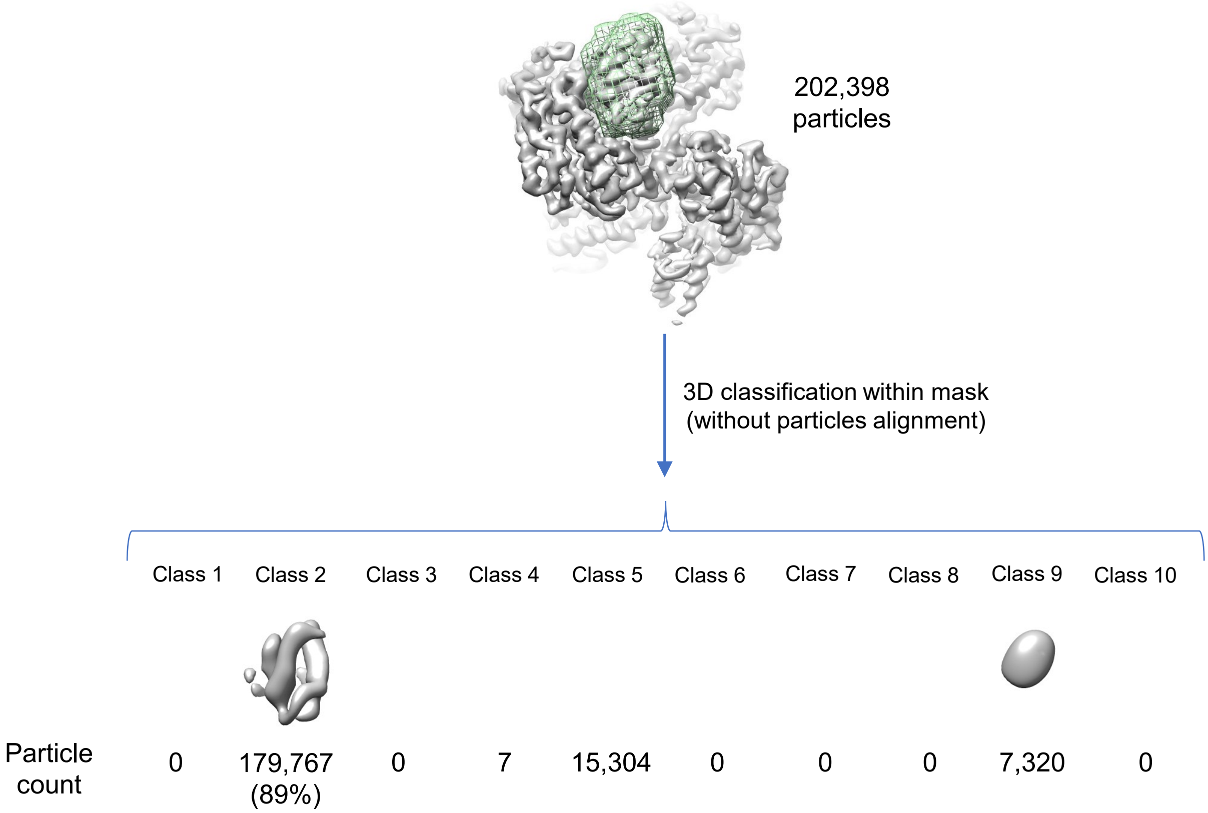

Supplementary Figure 2. Example of determination of the occupancy of the intermediate domain docked into the substrate entrance tunnel of the C_t domain using 3D classification within a mask. The estimated occupancy is 89%.

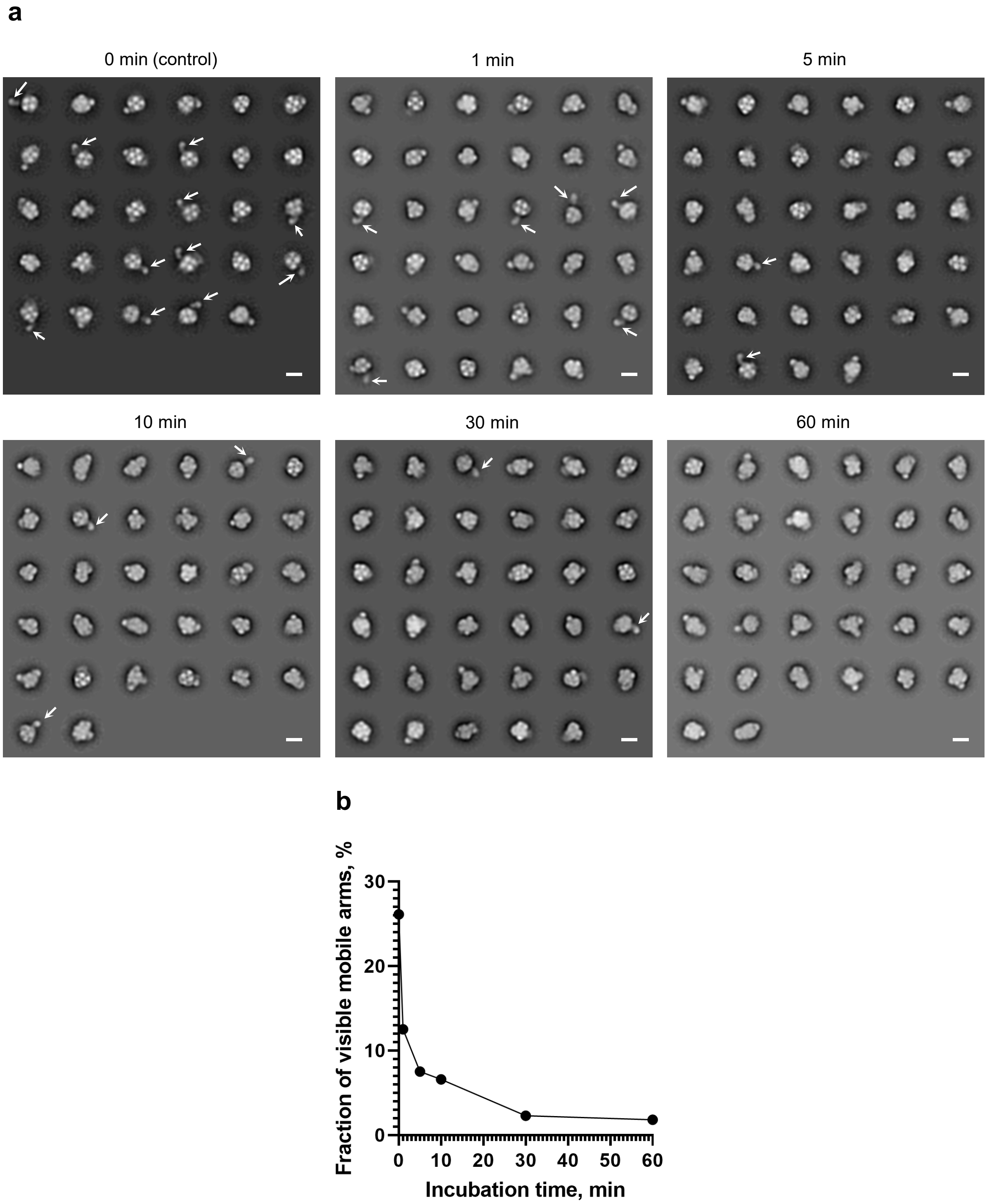

Supplementary Figure 3. Cross-linking with glutaraldehyde immobilizes N-terminal domains of ALDH1L1 in non-random positions. **a**: Negative-stain 2D class averages of ALDH1L1 treated with 0.1% glutaraldehyde at 4°C for 0-60 min. Arrows point to visible mobile arms (N-terminal domains). **b**: Results of quantification of resolved mobile arms versus incubation time.

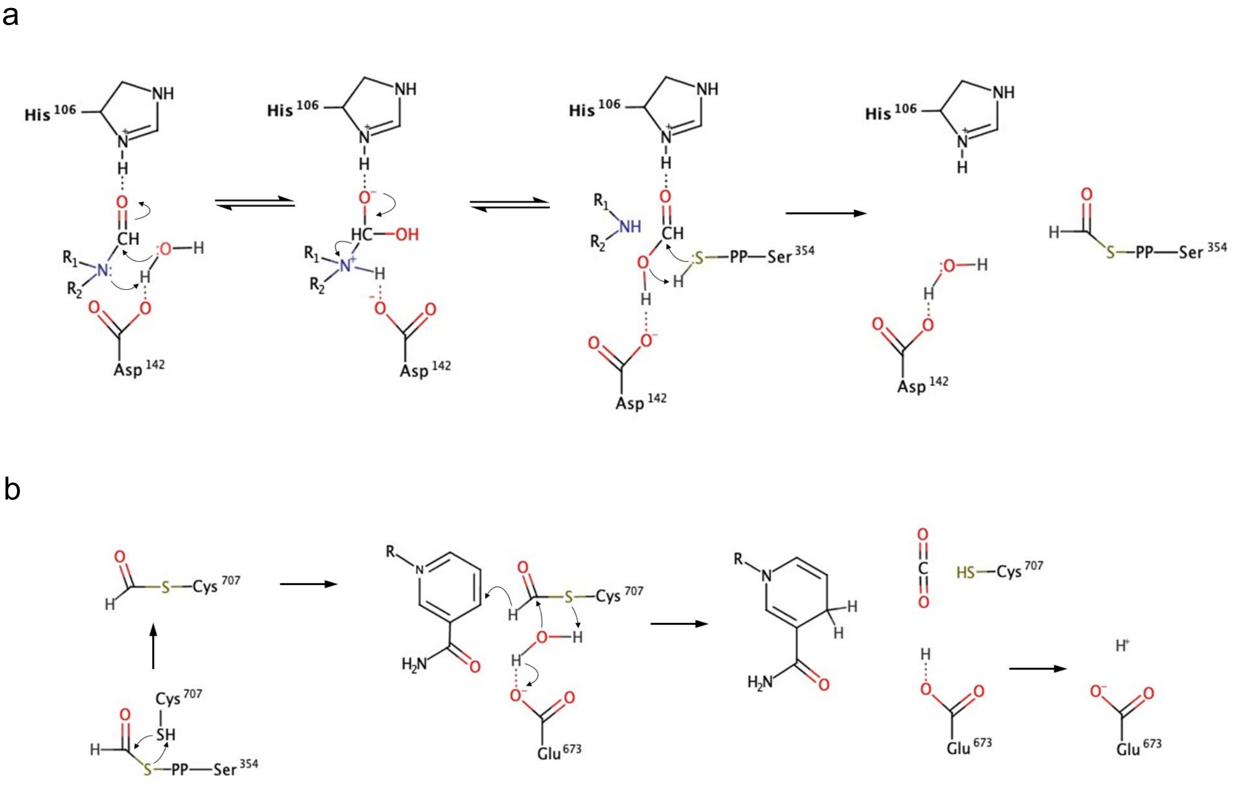

Supplementary Figure 4. Proposed two-step mechanism of ALDH1L1 catalysis. **a:** Formyl transfer from 10-formylTHF to the 4'-phosphopantetheine (4'-PP) arm in the folate-binding hydrolase domain (N_t-domain). Asp142 activates a water molecule for hydrolysis of the bond between the carbonyl carbon of the formyl group and N^{10} of 10-formylTHF while His106 helps to orient the carbonyl group. Formate remains bound in the active site until the 4'-PP arm swings in. The sulfur of the 4'-PP attacks the carbon of the formate, forming a covalent bond, with simultaneous transfer of the proton from the sulfhydryl group to the oxygen, thus releasing a water molecule. Overall, the water molecule that participates in this step is restored upon the transfer of the formyl group to 4'-PP. **b:** Formyl oxidation in the C-terminal ALDH domain (C_t-domain). The formyl group is first transferred from the 4'-PP moiety to the sulfur atom of catalytic Cys707 and then is oxidized to CO_2 in the presence of $NADP^+$; a water molecule activated by Glu673 participates in this step.

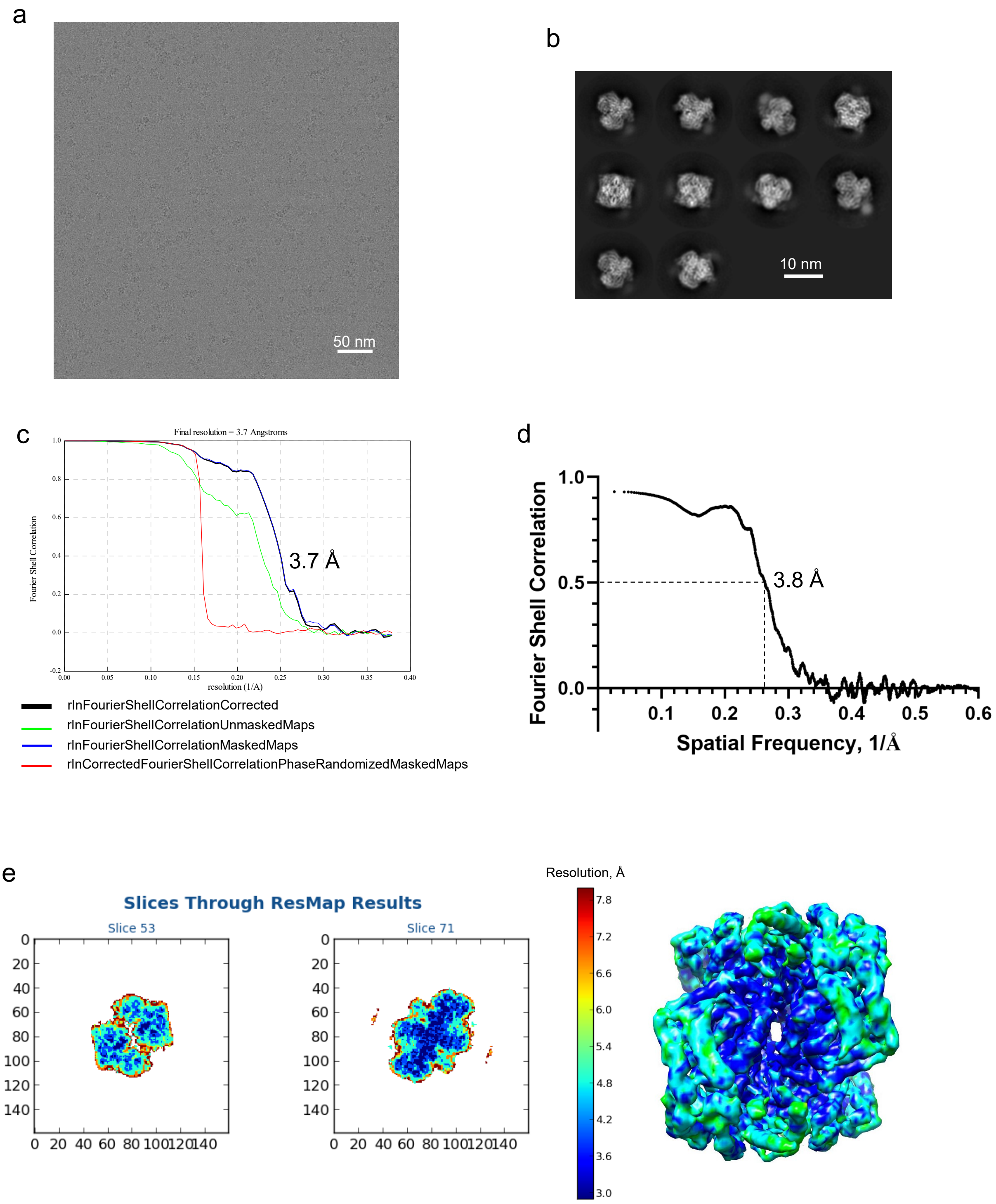

Supplementary Figure 5. Details of Cryo-EM reconstruction of ligand-free ALDH1L1. **a**: A representative micrograph. **b**: Representative 2D class averages. **c**: Resolution estimation and validation plots generated by Relion. **d**: Fourier shell correlation curve between the cryo-EM map and the molecular model. **e**: Local resolution estimation with ResMap. *Left*: slices through the map at two different levels. *Right*: map colored according to local resolution.

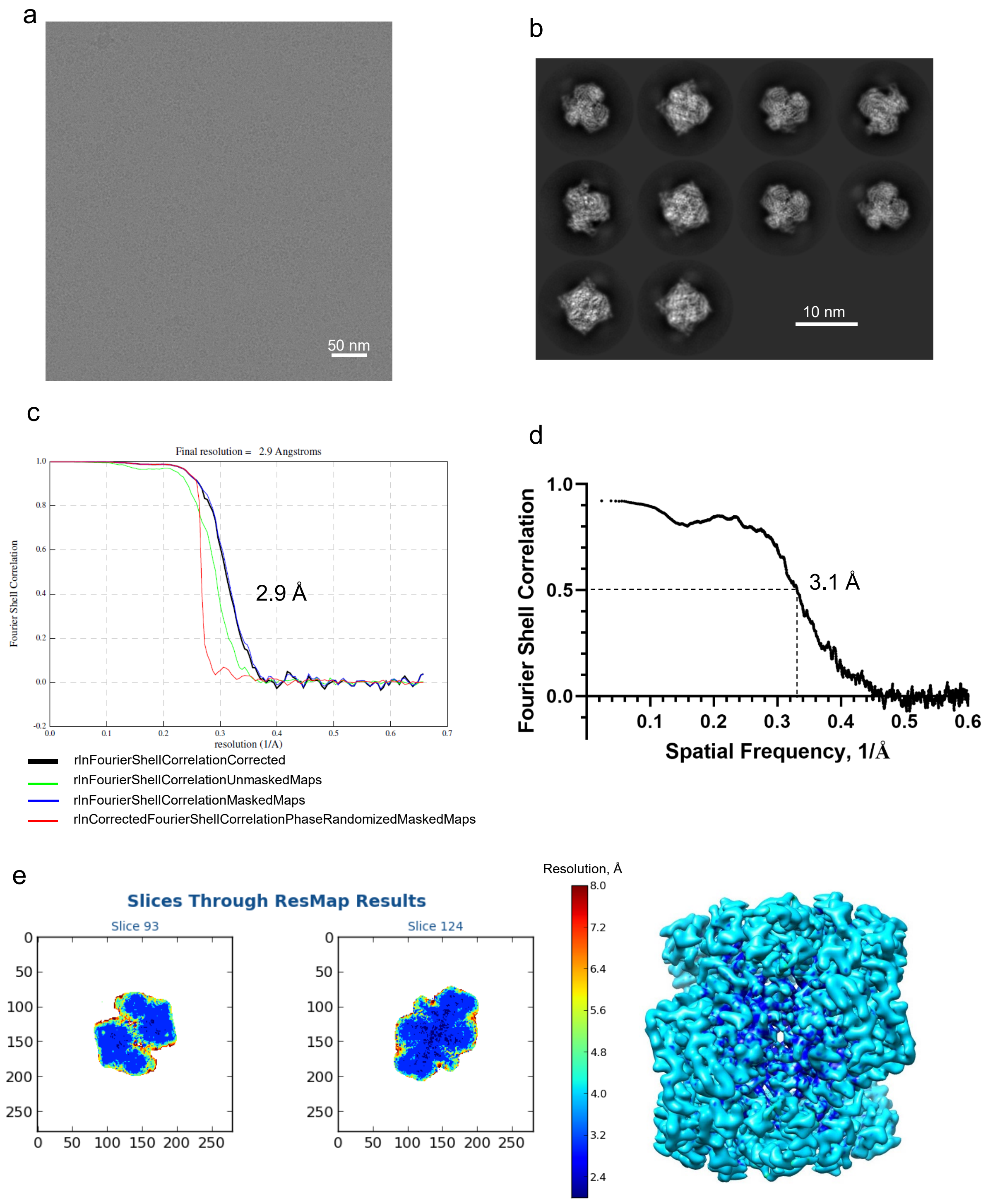

Supplementary Figure 6. Details of Cryo-EM reconstruction of ALDH1L1 in complex with NADP$^+$. **a**: A representative micrograph. **b**: Representative 2D class averages. **c**: Resolution estimation and validation plots generated by Relion. **d**: Fourier shell correlation curve between the cryo-EM map and the molecular model. **e**: Local resolution estimation with ResMap. *Left*: slices through the map at two different levels. *Right*: map colored according to local resolution.

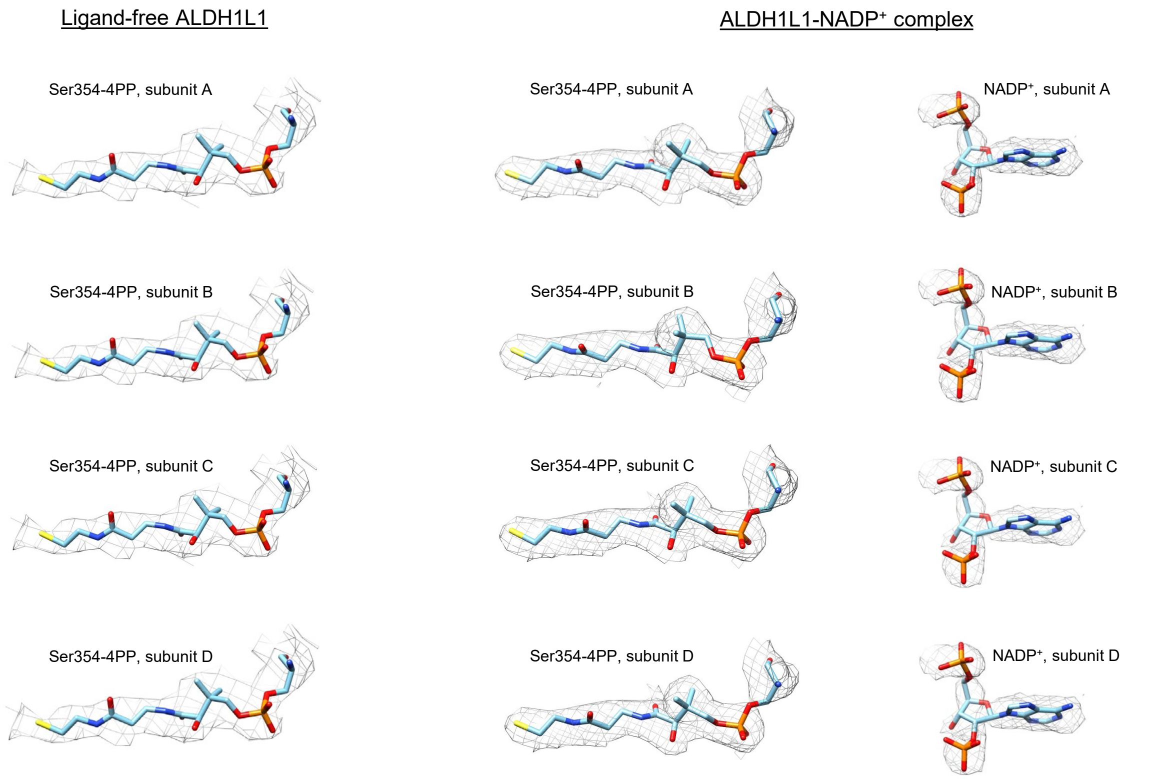

Supplementary Figure 7. Cryo-EM density for the 4'-phosphopantetheine (4PP) arms and $NADP^+$ molecules in the cryo-EM structures of ligand-free ALDH1L1 and ALDH1L1 in complex with $NADP^+$. For $NADP^+$, only density for the adenine, ribose and two phosphates was present.
